# Global Availability of Antivenoms: The Relevance of Public Manufacturing Laboratories

**DOI:** 10.3390/toxins11010005

**Published:** 2018-12-24

**Authors:** José María Gutiérrez

**Affiliations:** Instituto Clodomiro Picado, Facultad de Microbiología, Universidad de Costa Rica, San José 11501-2060, Costa Rica; jose.gutierrez@ucr.ac.cr; Tel.: +506-2511-7865

**Keywords:** antivenoms, snakebite envenoming, public laboratories, availability, preclinical efficacy

## Abstract

Snakebite envenoming is a serious global public health problem, and international initiatives, under the coordination of the World Health Organization and its regional offices, are being developed to reduce the impact of this neglected tropical disease. The global availability of safe and effective antivenoms is one of the key aspects in this global strategy. This review discusses the role of public antivenom manufacturing laboratories for ensuring the supply of antivenoms. The difficulties faced by public laboratories are discussed, together with some tasks that need to be implemented for strengthening them. In addition, the concept of ‘redundancy’ in the supply of antivenoms is emphasized, as a way to cope with the risks associated with the provision of antivenoms by few manufacturers. In general, the public sector should play a leading role, in antivenom availability and other aspects as well, within the global struggle to reduce the mortality and morbidity caused by snakebite envenoming.

## 1. Introduction

Snakebite envenoming is a highly relevant public health problem on a global basis that kills and maims several hundred thousand people every year [1,2]. It has been classified by the World Health Organization (WHO) within the group of Neglected Tropical Diseases (NTDs) [2], which predominantly affect the most impoverished populations in sub-Saharan Africa, Asia, Latin America, and parts of Oceania. As such, snakebite envenoming is a disease of the poor [3,4]. For many decades snakebite envenoming lingered without attention from health authorities, research agendas, the pharmaceutical industry, and health advocacy groups. Fortunately, owing to the concerted action of many stakeholders, in the last years there has been a growing global awareness on the impact of this tropical disease, and significant steps forward have been taken for attending to it. The adoption of snakebite envenoming as a NTD by the WHO [5] has resulted in the conformation of a WHO working group that is preparing a strategic plan with short-, medium-, and long-term objectives aimed at drastically reducing its impact [6]. This WHO strategic plan will be presented shortly to the international community.

As a further development, the 71st World Health Assembly, celebrated in Geneva in May 2018, adopted a resolution proposed by 22 countries from five continents, with the leadership of Costa Rica and Colombia, aimed at addressing the burden of snakebite envenoming on a global basis. This historical resolution urges member states to take immediate actions on several lines, with the aim of reducing the burden of this disease. These include: (a) Assessing the magnitude of the problem on a global basis; (b) improving antivenom availability, accessibility, and affordability; (c) promoting transfer of knowledge on this topic between member states; (d) integrating control of snakebite envenoming with efforts to control other diseases; (e) improving access to treatment and rehabilitation services to affected people; (f) providing training to health professionals in the diagnosis and treatment of envenomings; (g) supporting research activities on several aspects of snakebite envenoming; (h) promoting community awareness through culturally contextualized public campaigns; and (i) fostering of cooperation and collaboration between member states, the international community, and other relevant stakeholders. Likewise, this resolution established a number of requests to the Director General of the WHO on this health issue (see the resolution at [7]).

This multi-component and multi-stakeholder approach integrates a diverse set of actions and actors, through a network of synergies within a frame of solidarity and cooperation. One of the most pressing needs for effectively implementing this strategy has to do with the availability, accessibility, and affordability of safe and effective antivenoms on a global basis, a complex task in the light of the inherent difficulties involved. Antivenoms are included in the WHO list of essential medicines [8]. Historically, antivenoms have been out of the mainstream of pharmaceutical development and manufacture, and there has been a chronic deficit in antivenom availability in various regions of the world, most notably in sub-Saharan Africa [4,9,10]. Moreover, the regulation of antivenom manufacture and distribution has been deficient in many countries, hence facilitating the introduction of ineffective antivenoms that lack proper preclinical assessment and quality controls. Adding to this scenario is the fact that, even though antivenoms are purchased by public health systems in many countries, they are often inaccessible in remote rural regions of sub-Saharan Africa, Asia, and Latin America, where the majority of snakebites occur [4,11]. Therefore, a vicious cycle of antivenom market decline has occurred, driven by the lack of resources for antivenom procurement and the low confidence of antivenom safety and efficacy. This has caused a drop in the demand and sales of these products, which forces manufacturers to increase the price of antivenoms, hence contributing to a reduced demand [12,13,14].

There is an urgent need to increase the availability of antivenoms globally, by enhancing the capacity of current antivenom producers, as well as promoting the introduction of new manufacturers to this field. In parallel, it is necessary to improve the standards of antivenom production, by revising the design of antivenoms, implementing Good Manufacturing Practices (GMPs), strengthening the national regulatory agencies, and ensuring an appropriate characterization of the neutralizing efficacy and safety of antivenoms through preclinical and clinical research [15,16]. In this context, the concept of ‘redundancy’ in antivenom availability is highly relevant. This means that, in order to ensure that antivenoms are available to public health systems, several antivenoms of proven efficacy and safety should be offered in every region. This will be a safeguard in the event that one or several manufacturers fail to provide the required volume of antivenom. This requires a variety of interventions tailored to the needs of manufacturers, both public and private, in order to improve their performance. The present review focuses on the tasks to strengthen the public antivenom-manufacturing laboratories as key stakeholders in the global strategy to confront snakebite envenoming.

## 2. Exploring the Universe of Antivenom Manufacturing Laboratories

**Figure 1:**
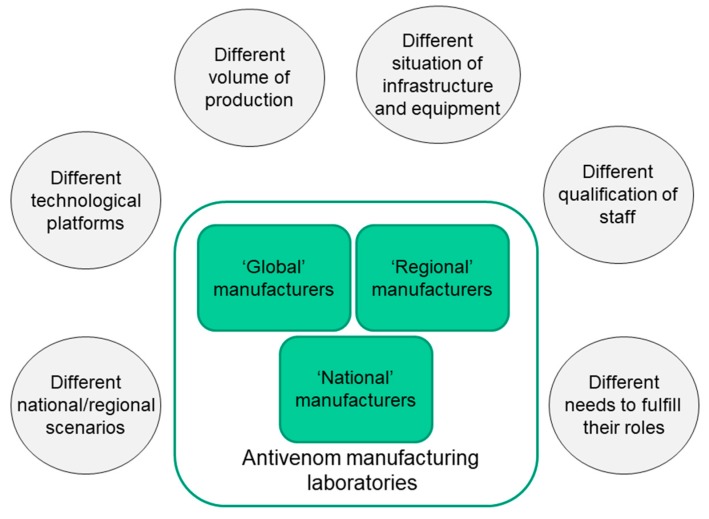
The heterogeneous global landscape of antivenom manufacturers. There is a large heterogeneity in the characteristics of antivenom manufacturing laboratories in terms of volume of production, range of geographical distribution of antivenoms, and technical and infrastructural features. ‘National’ manufacturers produce for their own countries, whereas ‘regional’ manufacturers produce for a group of countries in their own regions. ‘Global’ manufacturers produce and distribute antivenoms to several continents. Within this universe, there are private and public laboratories. Reproduced from Ref. [11], copyright 2012, Elsevier.

According to the WHO webpage on antivenoms [17], there are 46 laboratories which produce animal-derived antivenoms in the world, although it is likely that this number has changed over the last years. The majority of these laboratories are located in Asia and the Americas, followed by Europe, whereas fewer manufacturers are located in Africa, and one is in Australia. There is a large heterogeneity between these laboratories in the scale of their production and in the characteristics of antivenoms. Some manufacturers, mostly located in the public sector, produce antivenoms only for their own countries (‘national’ manufacturers), whereas others distribute antivenoms not only for their country, but also for neighboring countries (‘regional’ manufacturers), and few laboratories produce and distribute antivenoms to countries located in several continents (‘global’ manufacturers) [11] (Figure 1). Of the 46 antivenom manufacturers listed in the WHO webpage, 31 correspond to public institutions and 15 are private companies. Although, with some exceptions, private laboratories produce high volumes of antivenoms, some public laboratories also do so, and there are several public institutions that manufacture relatively low volumes that fulfill the needs of their own countries.

### 2.1. Technical Heterogeneity in Antivenom Manufacture

The heterogeneity of this universe involves not only the volume of production and scope of distribution, but also differences in some technical aspects of antivenom design and production. Most manufacturers use horses for immunization, although one uses sheep [18], and another one works with donkeys [19]. Some antivenoms are ‘monospecific’, i.e., they are produced by immunizing animals with the venom of one species, whereas most antivenoms are ‘polyspecific’, i.e., are prepared by using venom from two or more snake species. The latter have the advantage that the identification of the offending snake is not necessary for selecting the antivenom to be used, and often a syndromic approach for diagnosis is followed [20]. The majority of antivenoms include F(ab’)_2_ fragments as the active substance, by fractionating hyperimmune plasma with a combination of pepsin digestion and salting out procedures. Other products are made of whole IgG molecules, usually obtained by caprylic acid precipitation of non-immunoglobulin plasma proteins, and one manufacturer produces Fab antivenoms by digesting the immunoglobulins from ovine plasma with papain [18]. Products also vary in the excipients and preservatives used and in the final presentation, as some are liquid whereas others are freeze-dried, with the consequent implications in terms of storage and transportation, since liquid antivenoms require a cold chain system [21].

### 2.2. Additional Aspects of a Heterogeneous Landscape

Besides these variations in the design, and in the immunological and physico-chemical characteristics of antivenoms, there are marked differences in the situation of the infrastructure, laboratory equipment, qualification of technical and professional staff, implementation of GMPs, innovation, and in-process and final quality control of the products (Figure 1). Some manufactures have made significant investments in their infrastructure and equipment, as well as in the training and upgrading of the qualifications of their personnel, fully complying with GMPs. In contrast, other laboratories have lagged behind due to limited innovation and investment in infrastructure and equipment, and deficient training programs for their staff. Other manufacturers, in turn, lie in an intermediate position between these extremes. The economic hurdles associated with antivenom production, related to uncertain markets, poor economies of scale, and lack of innovation and investment, place this manufacturing field in a rather vulnerable scenario that demands urgent actions at various levels, with the involvement of the WHO and its regional offices, public health foundations and donors, investors, research and national regulatory agencies, and manufacturers themselves.

## 3. The Situation of Public Antivenom Manufacturing Laboratories

The public health arena has witnessed significant shifts in the conceptualization of health in the last five decades. The concept of health as a ‘commodity’, within the context of a market-driven economy, has been fostered, together with trends in developing countries’ governments to reduce the public expenditure in health and other social services (for a detailed description of the ups and downs of global public health in the last decades, see Ref. [22]). Nevertheless, the ideal of ‘health for all’ has continued to permeate the global health agenda, and has made its way to relevant global initiatives, such as the Millennium Development Goals (MDGs) [23] and the 2030 Agenda for Sustainable Development [24], launched by the United Nations. ‘Leave no one behind’ is a motto of the United Nations that reflects these goals [25].

In this global context, public antivenom manufacturing laboratories have faced significant challenges in the last decades. Public manufacturers of antivenoms were created in many countries during the past century and represent invaluable institutional assets, constituting scientific, technological, and public health patrimonies. In the Americas, the first public institution in charge of manufacturing antivenoms was Instituto Butantan, created in 1901 in São Paulo, Brazil [26]. Then, several public laboratories were created in Brazil, Argentina, Uruguay, Bolivia, Peru, Ecuador, Colombia, Venezuela, Costa Rica, and Mexico (see Ref. [17]). They have provided a long standing contribution to the control of snakebite envenoming through the supply of antivenoms for their own countries and, in some cases, for other countries in the region. Nevertheless, in the previous decades, many of these laboratories have faced serious challenges to keep up with the necessary investment for their development, and some of them currently have problems at various levels, in large part owing to the limitations suffered by public health institutions, as described above.

**Figure 2:**
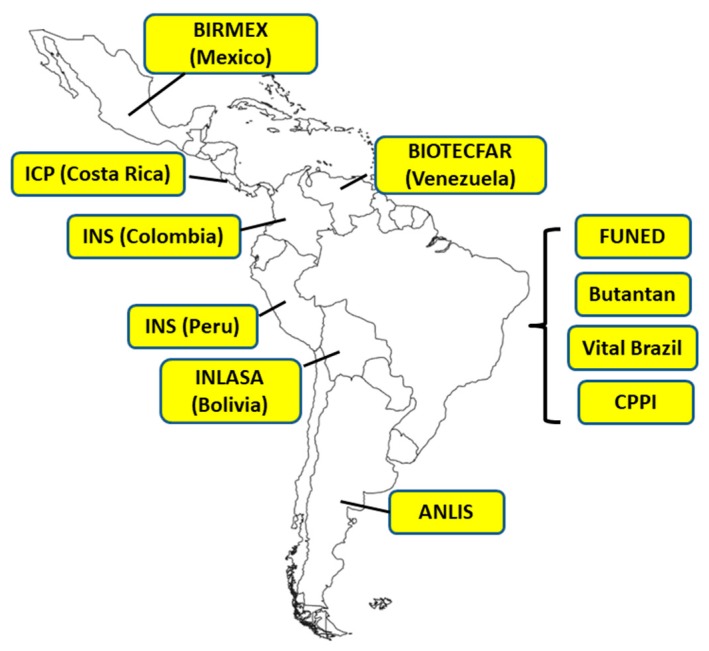
Network of public antivenom manufacturing laboratories in Latin America. Public antivenom manufacturing laboratories exist in Argentina (ANLIS-Administración Nacional de Laboratorios e Institutos de Salud); Brazil (FUNED, Fundação Ezequiel Dias; Instituto Butantan; Instituto Vital Brazil; CPPI, Centro de Pesquisa e Produção em Imunobiológicos); Bolivia (INLASA, Instituto Nacional de Laboratorios de Salud); Peru (INS, Instituto Nacional de Salud); Colombia (INS, Instituto Nacional de Salud); Venezuela (BIOTECFAR, Universidad Central de Venezuela); Costa Rica (ICP, Instituto Clodomiro Picado, Universidad de Costa Rica); Mexico (BIRMEX, Laboratorio de Biológicos y Reactivos de México). In Ecuador, antivenom manufacture was halted several years ago, but plans are underway by INSPI (Instituto Nacional de Salud Pública) to re-start antivenom manufacture in the future.

Several workshops of public antivenom manufacturing laboratories have been organized in Latin America during the last decade to discuss the situation of antivenom production in the region (see for example Ref. [19]), and exchanges of information have been made between Latin American groups and some public laboratories in Asia. Figure 2 depicts the distribution of public antivenom manufacturing laboratories in Latin America. A number of issues regarding the needs of these laboratories have been raised during these meetings and exchanges. Attention to these aspects should pave the way for a systematic improvement of public laboratories to the regional and global availability of safe and effective antivenoms.

### 3.1. Limitations in Research and Development

Many public laboratories in charge of antivenom manufacture lack innovation through research and development. Many have not modified the basic technological platform for plasma fractionation and for in-process and final quality control. An example is the improvement in the design of the most adequate combinations of venoms for immunization. Most of the combinations currently used were selected decades ago on the basis of rather empirical criteria. In the last decade there has been an enormous volume of knowledge generated on the biochemistry of snake venoms, including their proteomics, as well as on the cross-reactivity of antivenoms, by using neutralization tests and immunochemical methods, particularly antivenomics [15,27,28]. Likewise, the taxonomy of several medically relevant snake species has changed. This wealth of information should be harnessed to evaluate the design of the most appropriate venom mixtures used for antivenom production, and to establish novel combinations for new antivenoms to be developed. To attain this goal, it is necessary to build partnerships between antivenom-production laboratories and research groups in their own countries and abroad. An intense cross-talk between these partners should result in improvements in antivenom design. Likewise, the field of preclinical evaluation of antivenoms has grown and now consists of a toolkit of assays to evaluate the neutralizing scope of antivenoms, and also to substitute in vivo tests for in vitro assays within the context of the 3Rs principle (Replacement, Reduction, Refinement) [29].

The immunization protocols require a thorough revision and innovation in aspects such as selection of venom doses to be injected, method of injection (i.e., single site or multisite), use of novel adjuvants, schedule of venom injections as to avoid the immunosuppressive effects that some venoms exert over others, and a more detailed control of animal health status during immunization. Moreover, the protocols for animal bleeding can be improved through innovation in aspects such as the use of automated plasmapheresis equipment.

There are plenty of possibilities to innovate in the technologies used for hyperimmune plasma fractionation in order to generate products of higher purity and better physico-chemical characteristics. Plasma processing groups should evaluate the steps in their fractionation protocols in order to optimize them. As an example, the introduction of caprylic acid fractionation instead of ammonium sulfate precipitation in the preparation of whole IgG antivenoms should be considered [30]. Likewise, substituting gravity filtration for centrifugation or in-depth filtration systems for separating precipitates along the process has to be considered, together with the use of tangential filtration and the introduction of viral removal and inactivation steps. The design of the freeze-drying program also requires innovation as to adapt it to the formulation of each antivenom. To achieve these goals, research and development groups in production laboratories should be created, and a close association with research institutions working on protein purification can bring positive outcomes, particularly with groups in the Chemical Engineering and Biotechnology fields.

### 3.2. The Need to Invest in Infrastructure and Equipment

There has been a notorious lack of investment in infrastructure and equipment in many public antivenom-manufacturing laboratories. Owing to the growing financial limitations of public institutions and governments along the last decades, some of these laboratories have lagged behind in the need to update their infrastructure and to replace old equipment, with the consequent negative impact in antivenom production. Likewise, maintenance programs for equipment are limited. This has placed a number of laboratories at the brink of shutting down their operation. The solution to this situation demands creative political decisions in order to reinvigorate the financial support for these public laboratories by governments. There are notorious contrasts in the political will of governments of countries in the support of antivenom laboratories. In addition, institutions need to consider the development of public–private partnerships that may contribute to the upgrading of these laboratories. Likewise, international funding agencies in the field of public health should consider supporting public antivenom-manufacturing laboratories of long-standing tradition. The strengthening of public laboratories in various parts of the world is a safeguard for guaranteeing antivenom availability in the midst of ups and downs associated with a market economy.

### 3.3. Improvement in Training and Qualification of Staff

Public antivenom manufacturing laboratories are often understaffed, as a consequence of restrictive policies in governmental institutions. Moreover, there is a deficit in programs aimed at the permanent training and qualification of the existing personnel, which limits innovation. The possibilities for technical and professional staff to attend workshops, courses, and symposia are scant. This trend has to be reverted by establishing permanent training programs, adapted to the needs of the staff of manufacturing laboratories. Such training activities can be coordinated with local universities and other public and private institutions, and funding must be allocated for these activities. Likewise, seminars and group discussions of advances in the field and novel technological developments need to be organized, and an innovative environment should be stimulated within these laboratories. In Latin America, a group of public laboratories have integrated a regional network in which regular workshops have been organized. These activities have had a significant impact in the overall improvement of antivenom availability in the region (see for example Refs. [19,31]). In summary, a culture of creativity and innovation, associated with permanent improvement of the qualification of staff, is mandatory for the strengthening of these manufacturing facilities.

One aspect that requires urgent attention in these institutions is the implementation of GMPs at all levels in the chain of antivenom production. For this, professional and technical staff should receive training based on international guidelines, including the *WHO Guidelines for the Production, Control and Regulation of Snake Antivenom Immunoglobulins* [18], and the directive councils of these laboratories should support these activities and the necessary investments to attain the fulfillment of GMPs. In this regard, the National Regulatory Agencies have a role to play by having a close coordination with manufacturing laboratories, providing advice and guidance along the regular inspections of the facilities and protocols. Likewise, the stability of key technical staff is of utmost importance for guaranteeing the sustainability of the projects. The political upheavals often related with changes in the governments and in the directorship of manufacturing institutions should not affect the technical continuity of the projects.

### 3.4. Promoting International Cooperation

The tasks described could be achieved more successfully within a frame of international cooperation. To this end, the WHO and its regional offices have a leading role to play by establishing permanent programs for improvement of antivenom production and quality control, based on the diagnosis of the situation of these laboratories. These programs can involve regional workshops and seminars, as well as a variety of activities aimed at enhancing the capacity of manufacturers. At the same time, a dynamic networking activity should be fostered between manufacturing and quality control laboratories, regulatory agencies and research groups in universities and other institutions. For this, the use of information and communication technologies offers great possibilities. In Latin America, the network of public antivenom manufacturing laboratories has established an informal dynamics of inter-group consultations on specific aspects, which has become a useful mechanism for trouble shooting in antivenom production. Technology transfer projects and cooperation contribute to the upgrading of antivenom manufacture in public laboratories.

### 3.5. Improving Managerial Capacity

The deficit in resources and personnel is often associated with weak managerial practices which hamper the productivity of these laboratories. Aspects such as estimation of production costs, purchasing and maintenance programs, administration of inventories, organization of the flow of activities, efficient allocation of staff in the production departments, distribution and marketing of antivenoms, and quality assurance programs need to be introduced, evaluated, and improved aiming at increasing the efficiency of antivenom manufacture. Likewise, preparation of strategic development plans and their follow up have to be considered. Overall, modern managerial and organizational procedures should be implemented in order to maximize the use of available resources. The appropriate selection of personnel in charge of managerial roles is of key relevance, as well as the differentiation of the profiles and suitability of people selected for managerial roles and those in charge of the more technical functions in antivenom manufacture.

## 4. A Lesson Not to be Forgotten: The Case of Brazil

A crisis that took place in Brazil in the 1980s vividly illustrates the relevance of public institutions, including antivenom manufacturers, in ensuring the availability of antivenoms for health systems and the overall attention to snakebite envenoming. Antivenom production in Brazil started with the foundation of Instituto Butantan, in 1901, under the leadership of Vital Brazil Mineiro da Campanha. Then, in 1907 the Fundação Ezequiel Dias (FUNED) was created in Minas Gerais and, in 1919, Vital Brazil moved to Niteroi, State of Rio de Janeiro, to establish antivenom production at Instituto Vital Brazil. These three public manufactures developed over the next decades; however, at some point in the second half of the 20th century there was a reduction in public investment and innovation in this antivenom-manufacturing industry. By the 1970s, a multinational pharmaceutical company (Syntex do Brasil) was providing the majority of vaccines and antivenoms being used in Brazil. In 1973, the Brazilian government created the National Immunization Program, to take charge of the national immunization programs and the provision of antivenoms as well. This was followed in 1980 by the creation of the Instituto Nacional de Controle de Qualidade em Saúde (INCQS-National Institute for Quality Control in Health). INCQS detected important flaws and deficiencies in the biological products, including antivenoms, and the production of immunobiologicals was halted. As a consequence, Syntex do Brasil decided to permanently stop the manufacture of antivenoms and vaccines, creating a serious national crisis in the supply of these products, since the public laboratories at that point had limitations and were unable to cope with the national demand [26,32].

In this context, the Brazilian federal government decided to create the National Self-Sufficiency Program in Immunobiologicals, with the goal of making this country self-sufficient in the provision of these essential vaccines and antivenoms, a wise political decision that has had long-term consequences for public health in Brazil. As a follow-up step, a committee for the study of envenomings and the production and testing of antivenoms was created. It was responsible for defining the most adequate combination of venoms to be used in the manufacture of antivenoms, the standardization of the potency tests for assessing antivenom efficacy, and the establishment of reference venoms for the most important snake species, to be used by manufacturers and quality control laboratories. Simultaneously, in 1985, a large investment by the federal government was implemented with the goal of making Brazil self-sufficient in antivenoms and several vaccines. This allowed the national public manufacturing laboratories to develop new infrastructure and significant technological improvements in their facilities. Behind this policy lay the belief that the country should be self-sufficient in the manufacture and distribution of life-saving antivenoms and vaccines through the work of public laboratories.

In addition, a national surveillance system for documenting envenomings by bites and stings of animals was developed, together with a national policy of antivenom and vaccine distribution in charge of the National Program of Immunization. Since 1986, antivenom acquisition is centralized by the Brazilian Ministry of Health, and distribution is decentralized to the states and municipalities, which allows free-of-charge availability of antivenom treatment. The production of antivenoms at a national level is coordinated between the Ministry of Health and the four manufacturing laboratories (Instituto Butantan, FUNED, Instituto Vital Brazil, and the Centro de Pesquisa e Produção em Imunobiológicos (CPPI-Center for Research and Production of Immunobiologicals). Likewise, an active research program in public universities and institutes was strengthened, including basic research in the biochemistry, toxicology, and immunology of animal venoms, as well as epidemiological and clinical studies on envenomings and their treatment. In other words, the country decided to control the problem of envenomings by animal bites and stings through a well concerted and supported public program which has consolidated over the decades, and has made this country self-sufficient in the provision of antivenoms and some vaccines, and a leader in toxinological research. For a detailed account on these developments, see Refs. [26,32], and references therein.

This brief historical account provides several lessons: (a) The dependence of a single manufacturer for the provision of an essential medicine, such as antivenom, puts the public health system of a country in a vulnerable position, as this Brazilian experience shows. (b) The public health authorities and, in general, governments should have a leading role for ensuring the availability and accessibility of antivenoms in public health systems. (c) The concept of ‘redundancy’ in the provision of antivenoms is very well illustrated in this historical example. Brazil has four public laboratories in charge of antivenom manufacture, with different scales and volumes of production, which ensure a steady national supply in close coordination with the Ministry of Health. The benefit of this redundancy is evident when, for technical reasons, one of these laboratories needs to temporarily stop production to carry out maintenance works or remodeling of infrastructure. In those circumstances, the other laboratories provide the necessary volume of antivenoms for the country’s needs. (d) The four laboratories have a close coordination in their activities under a frame of cooperation, hence contributing to the solution of problems that may arise in any of them. (e) This inter-laboratorial coordination with the Ministry of Health ensures that antivenoms manufactured by any laboratory have the same potency and quality control specifications, and all batches of antivenom are evaluated by the INCQS. In summary, the experience of Brazil represents a clear example of the impact of consistent public policies over decades for ensuring the national production and self-sufficient supply of antivenoms.

It must be stressed that no single strategy in the field of antivenoms can be recommended for different countries and regions, since the local contexts greatly differ, and appropriate solutions have to be devised for each situation. For example, the concept of redundancy may apply to the combination of private and public, as well as national or international, manufacturing laboratories, and the variable scales of needs and production may demand different approaches, i.e., some countries do not have the conditions to develop their own antivenom-producing laboratories, hence having to rely on manufacturers from abroad. However, the basic idea of redundancy and the need for a strong commitment of public institutions and governments for ensuring antivenom availability remain as key components in the global efforts to reduce the impact of snakebite envenoming. Likewise, ministries of health should have national regulatory agencies with the capacity of evaluate the safety and efficacy of antivenoms produced in the country or imported. Overall, the public sector has to play a leading role in this field, as well as in other aspects of the control of snakebite envenoming.

## 5. The Concept of Redundancy as Applied to Latin America and the Caribbean

The concept of redundancy in the availability of antivenoms is well illustrated in the case of Latin America and the Caribbean. Several polyspecific and few monospecific antivenoms are manufactured in the region, with two laboratories (one public and one private) in Argentina, Colombia, and Mexico, four public laboratories in Brazil, and one public laboratory in Bolivia, Peru, Venezuela, and Costa Rica [17,19]. In addition, a French private laboratory provides a monospecific antivenom for Martinique [33]. These antivenoms are generated by using variable mixtures of venoms for immunization. For example, the polyspecific antivenom manufactured in Costa Rica and currently used in all Central American countries and in Ecuador and Saint Lucia is prepared by a mixture of venoms of *Bothrops asper*, *Crotalus simus*, and *Lachesis stenophrys* [34]. On the other hand, the bothropic antivenom manufactured by Brazilian laboratories uses a mixture of venoms of *Bothrops jararaca*, *B. jararacussu*, *B. neuwiedi*, *B. alternatus*, and *B. moojeni* [34], whereas the bothropic antivenom produced in Peru is based on the immunization of horses with the venoms of *Bothrops atrox*, *B. barnetti*, *B. pictus*, *B. brazili*, and *Bothrocophias hyoprora* [34]. Other combinations of venoms are employed in the preparation of antivenoms in Argentina, Bolivia, Venezuela, and Mexico [34].

Despite the variations in the immunization mixtures, various preclinical studies on the neutralizing ability of antivenoms against a wide array of venoms from snake species of the genera *Agkistrodon*, *Atropoides*, *Bothrops*, *Bothriechis*, *Cerrophidion*, and *Porthidium* have demonstrated an extensive pattern of cross neutralization of several of these antivenoms [15,34,35,36,37,38,39]. In the case of venoms of high medical impact in the region, such as those of *Bothrops asper*, *B. atrox*, *B. jararaca*, *B. neuwiedi* (*diporus*), and *B. neuwiedi* (*mattogrossensis*), polyspecific antivenoms produced in Argentina, Brazil, Bolivia, Peru, Colombia, Costa Rica, and Mexico are effective in the neutralization of lethal, hemorrhagic, coagulant, and myotoxic effects of these venoms, albeit with different values of Median Effective Doses (ED_50_) [34,40]. Likewise, antivenoms prepared using venoms of different species of *Lachesis* in the immunizing mixtures are able to neutralize venoms of the four species of *Lachesis* [41]. Regarding *Crotalus* sp venoms, two different patterns of venom composition have been described: type I venoms have a predominance of hemorrhagic metalloproteinases over the neurotoxic PLA_2_ dimer crotoxin, whereas type II venoms has a predominance of crotoxin and low amounts of metalloproteinases [42,43]. Thus, antivenoms produced in Argentina, Brazil, Peru, Colombia, Venezuela, and Mexico using type II venoms are effective in the neutralization of neurotoxic *Crotalus* sp venoms in South America and Mexico, whereas antivenoms prepared by using type I venoms are effective in Central America and against non-neurotoxic venoms in North America [15,43,44,45,46].

This body of evidence gives support to the use of several polyspecific antivenoms in different countries in Latin America. Hence, in the cases of countries that do not manufacture their own antivenoms (Uruguay, Paraguay, Ecuador; the Guyanas, Panama, Nicaragua, El Salvador, Honduras, and Guatemala), or those that may have deficits in their own supplies of antivenoms, several products from other countries can be used, thus constituting a scenario of regional self-sufficiency in antivenom supply. There is a need to further expand the studies on the preclinical efficacy of antivenoms in Latin America as to have a more complete view of the neutralizing spectrum of all antivenoms available in the region. This will help ministries of health and the Pan American Health Organization (PAHO) to know in detail which products could be used in which countries; this, in turn, could lead to regional purchasing schemes which ensure the regional availability of antivenoms. For example, polyspecific antivenoms from various manufacturers have been used in Central American countries, as well as in Colombia, the Guyanas, Paraguay, and Ecuador. This is an example of redundancy in the production and provision of antivenoms, an experience that could be replicated in other regions of the world.

## 6. Public Laboratories and the Preparation of ‘Orphan’ Antivenoms

One niche where public antivenom manufacturers can play a key role in antivenom availability has to do with the manufacture of ‘orphan’ antivenoms, that is, antivenoms for the treatment of envenomings by species that inflict few, but potentially fatal, accidents; consequently, these antivenoms have a reduced market and low economic profitability. Examples are *Micrurus* antivenoms in the Americas, *Atractaspis* antivenoms in Africa and the Middle East, antivenoms against some ‘colubrid’ (*sensu lato*) venoms that can induce fatal accidents in Africa and Asia, and sea snake antivenoms in the Indo-Australian region. These and other ‘orphan’ antivenoms can be manufactured by public laboratories, under a non-for-profit scheme, and regional coordinated policies by the ministries of health and the regional offices of the WHO could be implemented in order to support their production and to organize distribution policies that ensure availability. Therefore, these antivenoms are relevant not only in the countries where they are manufactured and in those where related snake species are distributed, but they are also highly valuable in countries where exotic snakes are imported for private use, research, or exhibits, and antivenoms are required in the event of an envenoming.

An example was the development of a Pan-American *Micrurus* antivenom by Instituto Clodomiro Picado, with the support of PAHO, during the 1970s [47]. Although this project was discontinued, similar efforts should be implemented in Latin America to produce *Micrurus* antivenoms of wide neutralizing coverage, hopefully in the context of cooperative agreements between various laboratories. Likewise, antivenoms against the ‘colubrids’ *Dispholidus typus* and *Rhabdophis tigrinus,* and against sea snakes (Elapidae) are produced in South Africa, Japan, and Australia, respectively [17]. Owing to the limited market of these products, coordinated programs are required to facilitate their sustainable production and to have readily available national and regional stocks. Diverse stakeholders, including public antivenom laboratories, need to work in cooperative schemes to ensure the availability of these antivenoms.

## 7. The Manufacture of Antivenoms in Public Laboratories within the Global Landscape of the Efforts to Control Snakebite Envenoming

As stated in the Introduction, the global efforts to reduce the impact and suffering caused by snakebite envenomings demand a multi-component, multi-stakeholder strategy involving international and national public health authorities, the scientific and technological research community, antivenom manufacturers and quality control laboratories, national regulatory agencies, university schools in charge of medical and nursing education, local community organizations, non-governmental organizations (NGOs) and foundations that support health programs, health advocacy groups, and others, in order to achieve the goals included in the World Health Assembly’s resolution of May 2018 on snakebite envenomings and in the WHO strategic plan.

A key component in this global agenda has to do with the improvement in the availability, accessibility, and affordability of antivenoms. For this, antivenom manufacture must be re-invigorated by initiatives aimed at augmenting provision of safe and effective products. An innovative approach is necessary in order to stimulate a steady growth in antivenom supply by (a) increasing the number of manufacturers worldwide through a policy of incentives; (b) elevating the volume of production by current and new manufacturers; (c) improving the quality of antivenoms by international cooperation networks and technology transfer schemes; (d) strengthening the national regulatory agencies to ensure that only appropriate antivenoms are introduced; (e) establishing financial programs for the consolidation of antivenom manufacturers; (f) introducing novel purchasing schemes that would favor a more stable market and create stimuli for manufacturers to increase their production; and (g) generating international cooperation platforms between antivenom manufacturing laboratories, research groups, health advocacy groups and foundations, and national and regional health authorities to improve antivenom availability and accessibility. Likewise, an increased availability of these products should be linked to interventions for improving their accessibility in remote rural regions where envenomings occur, and to train health personnel in the correct diagnosis and management of snakebite envenoming.

To attain these objectives, and to follow the concept of redundancy in antivenom provision, an eclectic universe of manufacturers should be stimulated, including national, regional, and global producers with different scales of production and markets, involving public as well as private laboratories. Some antivenoms may be polyspecific and have a wide geographical range of efficacy, whereas others may have a more restricted regional scope, or be directed against the venom of a single snake species, depending on the particular needs of countries.

Within this general landscape, public antivenom manufacturing laboratories have a relevant role to play. The invaluable expertise developed in these laboratories must be harnessed and upgraded, but the lack of political and economic support for many of them during the last decades threatens their productivity and even their existence. This trend should be reverted by ingenious and innovative programs supported by governments and international agencies. As discussed in this work, public laboratories constitute a safeguard for public health systems for the provision of antivenoms, as they are prone to resist the uncertainties of the market-driven antivenom manufacture and distribution.

## References

[1] Gutiérrez J.M., Theakston R.D.G., Warrell D.A. (2006). Confronting the neglected problem of snake bite envenoming: The need for a global partnership. PLoS Med..

[2] Gutiérrez J.M., Calvete J.J., Habib A.G., Harrison R.A., Williams D.J., Warrell D.A. (2017). Snakebite envenoming. Nat. Rev. Dis. Primers.

[3] Harrison R.A., Hargreaves A., Wagstaff S.C., Faragher B., Lalloo D.G. (2009). Snake envenoming: A disease of poverty. PLoS Negl. Trop. Dis..

[4] Williams D.J., Gutiérrez J.M., Harrison R.A., Warrell D.A., White J., Winkel K.D., Gopalakrishnakone P. (2010). The global snake bite initiative: An antidote for snake bite. Lancet.

[5] Chippaux J.P. (2017). Snakebite envenomation turns again into a neglected tropical disease!. J. Venom. Anim. Toxins Incl. Trop. Dis..

[6] WHO Working Group on Snakebite Envenoming. http://www.who.int/snakebites/control/WHO_Working_Group_on_Snakebite_Envenoming/en/index1.html.

[7] Seventy First World Health Assembly Addressing the Burden of Snakebite Envenoming. http://www.who.int/neglected_diseases/mediacentre/WHA_71.5_Eng.pdf?ua=1.

[8] WHO Model List of Essential Medicines. http://apps.who.int/iris/bitstream/handle/10665/273826/EML-20-eng.pdf?ua=1.

[9] Theakston R.D.G., Warrell D.A. (2000). Crisis in snake antivenom supply for Africa. Lancet.

[10] World Health Organization (2007). Rabies and Envenomings: A Neglected Public Health Issue. Report of a Consultative Meeting.

[11] Gutiérrez J.M. (2012). Improving antivenom availability and accessibility: Science, technology, and beyond. Toxicon.

[12] Cycle of Antivenom Market Decline. http://www.who.int/snakebites/antivenoms/Cycle_of_antivenom_market_decline.pdf?ua=1.

[13] Brown N.I. (2012). Consequences of neglect: Analysis of the sub-Saharan African snake antivenom market and the global context. PLoS Negl. Trop. Dis..

[14] Habib A.H., Brown N.I. (2018). The snakebite problem and antivenom crisis from a health-economic perspective. Toxicon.

[15] Gutiérrez J.M. (2018). Preclinical assessment of the neutralizing efficacy of snake antivenoms in Latin America and the Caribbean: A review. Toxicon.

[16] Williams D.J., Habib A.G., Warrell D.A. (2018). Clinical studies of the effectiveness and safety of antivenoms. Toxicon.

[17] World Health Organization List of Antivenoms. http://apps.who.int/bloodproducts/snakeantivenoms/database/.

[18] World Health Organization Guidelines for the Production, Control and Regulation of Snake Antivenom Immunoglobulins. http://apps.who.int/medicinedocs/documents/s23324en/s23324en.pdf.

[19] Gutiérrez J.M., Higashi H.G., Wen F.H., Burnouf T. (2007). Strengthening antivenom production in Central and South American public laboratories: Report of a workshop. Toxicon.

[20] World Health Organization Guidelines for the Prevention and Clinical Management of Snakebite in Africa. http://apps.who.int/iris/bitstream/handle/10665/204458/9789290231684.pdf?sequence=1.

[21] León G., Vargas M., Segura Á., Herrera M., Villalta M., Sánchez A., Solano G., Gómez A., Sánchez M., Estrada R. (2018). Current technologies for the industrial manufacture of snake antivenoms. Toxicon.

[22] Basilico M., Weigel J., Motgi A., Bor J., Keshavjee S., Farmer P., Kim J.Y., Kleinman A., Basilico M. (2013). Health for all? Competing theories and geopolitics. Reimagining Global Health. An Introduction.

[23] The Millennium Development Goals Report 2015. http://www.un.org/millenniumgoals/2015_MDG_Report/pdf/MDG%202015%20rev%20.

[24] United Nations Transforming our World: The 2030 Agenda for Sustainable Development. https://undocs.org/A/RES/70/1.

[25] United Nations Development Program What Does it Mean to Leave no One Behind?. http://www.undp.org/content/undp/en/home/librarypage/poverty-reduction/what-does-it-mean-to-leave-no-one-behind-.html.

[26] Squaiella-Baptistão C.C., Sant’Anna O.A., Marcelino J.R., Tambourgi D.V. (2018). The history of antivenoms development: Beyond calmette and vital Brazil. Toxicon.

[27] Calvete J.J. (2011). Proteomic tools against the neglected pathology of snake bite envenoming. Expert Rev. Proteom..

[28] Gutiérrez J.M., Lomonte B., Sanz L., Calvete J.J., Pla D. (2014). Immunological profile of antivenoms: Preclinical analysis of the efficacy of a polyspecific antivenom through antivenomics and neutralization assays. J. Proteom..

[29] Gutiérrez J.M., Solano G., Pla D., Herrera M., Segura Á., Vargas M., Villalta M., Sánchez A., Sanz L., Lomonte B. (2017). Preclinical evaluation of the efficacy of antivenoms for snakebite envenoming: State-of-the-art and challenges ahead. Toxins.

[30] Rojas G., Jiménez J.M., Gutiérrez J.M. (1994). Caprylic acid fractionation of hyperimmune horse plasma: Description of a simple procedure for antivenom production. Toxicon.

[31] Gutiérrez J.M. (2016). Understanding and confronting snakebite envenoming: The harvest of cooperation. Toxicon.

[32] Fan H.W., Monteiro W.M. (2018). History and perspectives on how to ensure antivenom accessibility in the most remote areas in Brazil. Toxicon.

[33] Resiere D., Mébarbane B., Valentino R., Mehdaoui H., Thomas L. (2010). *Bothrops lanceolatus* bites: Guidelines for severity assessment and emergent management. Toxins.

[34] Segura Á., Castillo M.C., Núñez V., Yarlequé A., Gonçalves L.R.C., Villalta M., Bonilla C., Herrera M., Vargas M., Fernández M. (2010). Preclinical assessment of the neutralizing capacity of antivenoms produced in six Latin American countries against medically-relevant *Bothrops* snake venoms. Toxicon.

[35] De Roodt A.R., Dolab J.A., Fernández T., Segre L., Hajos S.E. (1998). Cross-reactivity and heterologous neutralization of crotaline antivenoms used in Argentina. Toxicon.

[36] Bogarín G., Morais J.F., Yamaguchi I.K., Stephano M.A., Marcelino J.R., Nishikawa A.K., Guidolin R., Rojas G., Higashi H.G., Gutiérrez J.M. (2000). Neutralization of crotaline snake venoms from Central and South America by antivenoms produced in Brazil and Costa Rica. Toxicon.

[37] Rojas E., Quesada L., Arce V., Lomonte B., Rojas G., Gutiérrez J.M. (2005). Neutralization of four Peruvian *Bothrops* sp. snake venoms by polyvalent antivenoms produced in Perú and Costa Rica: Preclinical assessment. Acta Trop..

[38] Estévez J., Magaña P., Chippaux J.P., Vidal N., Mancilla R., Paniagua J.R., de Roodt A.R. (2008). Étude des venins des principaux serpents venimeux de Guyane française e de leur neutralisation. Bull. Soc. Pathol. Exot..

[39] Gutiérrez J.M., Sanz L., Escolano J., Fernández J., Lomonte B., Angulo Y., Rucavado A., Warrell D.A., Calvete J.J. (2008). Snake venomics of the Lesser Antillean pit vipers *Bothrops caribbaeus* and *Bothrops lanceolatus*: A correlation with toxicological activities and I mmunoreactivity of a heterologous antivenom. J. Proteom. Res..

[40] Segura Á., Herera M., Vargas M., Villalta M., Uscanga-Reynell A., León G., Gutiérrez J.M. (2017). Preclinical efficacy against toxic activities of medically relevant *Bothrops* sp. (Serpentes: Viperidae) snake venoms by a polyspecific antivenom produced in Mexico. Rev. Biol. Trop..

[41] Madrigal M., Pla D., Sanz L., Barboza E., Arroyo-Portilla C., Corrêa-Neto C., Gutiérrez J.M., Alape-Girón A., Flores-Díaz M., Calvete J.J. (2017). Cross-reactivity, antivenomics, and neutralization of toxic activities of *Lachesis* venoms by polyspecific and monospecific antivenoms. PLoS Negl. Trop. Dis..

[42] Mackessy S.P., Hayes W.K., Bearman K.R., Cardwell M.D., Bush S.P. (2008). Venom composition in rattlesnakes: Trends and biological significance. The Biology of Rattlesnakes.

[43] Calvete J.J., Sanz K., Cid P., de la Torre P., Flores-Díaz M., dos Santos M.C., Borges A., Bremo A., Angulo Y., Lomonte B. (2010). Snake venomics of the Central American rattlesnake *Crotalus simus* and the South American *Crotalus durissus* complex points to neurotoxicity as an adaptive paedomorphic trend along *Crotalus* dispersal in South America. J. Proteom. Res..

[44] Arce V., Rojas E., Ownby C.L., Rojas G., Gutiérrez J.M. (2003). Preclinical assessment of the ability of polyvalent (Crotalinae) and anticoral (Elapidae) antivenoms produced in Costa Rica to neutralize the venoms of North American snakes. Toxicon.

[45] Saravia P., Rojas E., Arce V., Guevara C., López J.C., Chaves E., Velásquez R., Rojas G., Gutiérrez J.M. (2002). Geographic and ontogenic variability in the venom of the neotropical rattlesnake *Crotalus durissus*: Pathophysiological and therapeutic implications. Rev. Biol. Trop..

[46] Segura Á., Herrera M., Reta-Mares F., Jaime C., Sánchez A., Vargas M., Villalta M., Gómez A., Gutiérrez J.M., León G. (2017). Proteomic, toxicological and immunogenic characterization of Mexican west-coast rattlesnake (*Crotalus basiliscus*) venom and its immunological relatedness with the venom of Central American rattlesnake (*Crotalus simus*). J. Proteom..

[47] Bolaños R., Cerdas L., Abalos J.W. (1978). Venoms of coral snakes (*Micrurus* spp.): Report on a multivalent antivenin for the Americas. Bull. Pan Am. Health Organ..

